# Specific Identification of *Listeria monocytogenes* in Food Using a QCM Sensor Based on Amino-Modified Mesoporous SiO_2_ with Enhanced Surface-Active Capabilities

**DOI:** 10.3390/foods14234151

**Published:** 2025-12-03

**Authors:** Ziliang Fan, Miaomiao Li, Xingyu Wang, Haixia Zhou, Faraz Ahmed, Yongheng Zhu

**Affiliations:** College of Food Science and Technology, Shanghai Ocean University, Shanghai 201306, China; 18888820272@163.com (Z.F.); 17836914529@163.com (M.L.); 18616302670@163.com (X.W.); d250400135@st.shou.edu.cn (H.Z.)

**Keywords:** dendrimer-like silica nanoparticles with hierarchical pores, quartz crystal microbalance sensor, rapid detection, foodborne pathogens

## Abstract

*Listeria monocytogenes* (*LM*) poses a serious threat to food safety and public health. Current detection methods suffer from drawbacks such as expensive equipment, complex procedures, and time-consuming processes, highlighting the urgent need for a simple, rapid, accurate, and cost-effective detection approach. The bacterial metabolite 3-hydroxy-2-butanone (3H2B), due to its high abundance, can serve as a reliable biomarker for detection. Herein, ordered mesoporous silica nanoparticles (MSNs) were synthesized via a one-pot method and subsequently functionalized with APTES. The NH_2_-MSNs-2 exhibits extremely high sensitivity (768 Hz@50 ppm) and selectivity towards 3H2B due to its high specific surface area, abundant mesoporous structure, and weak chemical adsorption between amino groups and the 3H2B. The quartz crystal microbalance (QCM) sensor developed based on this material demonstrated outstanding performance in testing the contamination levels of *LM* in food. This study provides a solid foundation for further exploring the fundamental mechanisms of QCM sensors in the real-time, non-invasive detection of *LM*, while also demonstrating significant application potential in the field of food safety assurance.

## 1. Introduction

*Listeria monocytogenes* (*LM*), a significant pathogenic bacterium transmitted via food, possesses distinct physiological traits that facilitate its prolonged viability and multiplication under refrigeration conditions typically found in household settings [1]. Infections caused by this bacterium, known as listeriosis, pose significant health risks to vulnerable populations, including pregnant women, neonates, the elderly, and immunocompromised individuals, potentially leading to fatal complications such as sepsis and meningitis with considerable mortality rates [2]. Due to its psychrotrophic characteristic, *LM* sustains metabolic functions even at refrigeration temperatures approaching 0 °C. Consequently, refrigerated ready-to-consumer products, including prepackaged salads, can become transmission vehicles, thereby introducing a pervasive hazard into the food safety system [3]. In light of discoveries that dispute the axiom of refrigeration safety and highlight frailties in the cold chain, a suite of detection technologies is currently employed, ranging from traditional plate culturing and immunoassays (ELISA) to molecular diagnostics (PCR), as well as tools utilizing mass spectrometry and flow cytometry [4]. These approaches are nevertheless hampered by several practical constraints. High instrument expenses, demanding procedural steps, long waiting times for results, and the necessity of expert staff for accurate sample processing represent common challenges [5]. Accordingly, creating swift, non-destructive tools to detect *LM* contamination in ready-to-eat foods is of great significance. These innovations are crucial for improving risk management within food safety systems [6].

A key MVOC produced by *LM* through its metabolism is 3-Hydroxy-2-butanone (3H2B). The measured levels of this volatile marker directly reflect the bacterial load present in contaminated food samples [7]. The suitability of 3H2B as an ideal biomarker stem from its distinct physical and chemical attributes. The compound’s molecular polarity promotes strong adsorption on functionalized nanosubstrates via non-covalent interactions. Furthermore, its intermediate level of volatility is conducive to efficient collection from the headspace [8]. This biomarker-based strategy represents a shift from traditional assays that require microbial growth. It facilitates rapid, molecular-level screening for *LM* within the food cold chain [9], applicable to items like prepackaged salads and prepared meats (e.g., prepackaged salads, ready-to-eat meats). With high sensitivity, real-time output, and label-free detection, quartz crystal microbalance (QCM) sensors are establishing themselves as an ideal analytical platform for gaseous analytes. Their potential has thus made them a prioritized subject for investigation across both academic and commercial sectors [10]. This sensing approach relies on the piezoelectric behavior of quartz. A mass change occurs as gas molecules accumulate on the sensing film, thereby perturbing the crystal’s fundamental oscillation frequency. Monitoring this specific parameter allows for the quantitative assessment of the analyte present in the gas phase [11]. Regarding sensitive material selection, semiconductor metal oxides (e.g., SnO_2_, ZnO) have been extensively studied for their catalytic activity in redox reactions, but their high operating temperatures (200–400 °C) limit their use in low-temperature applications [12]. Polymer materials enable selective adsorption of specific gases through molecular imprinting techniques, though their long-term stability requires improvement [13]. The two-dimensional nature of graphene oxide, coupled with its high density of oxygen-containing groups, creates unique strengths in the capture of gaseous species. Conversely, its performance as an electrical conductor is easily influenced by the presence of water vapor in the ambient environment [14]. Silicon dioxide (SiO_2_), as a typical inorganic nanomaterial, occupies an important position in nanotechnology due to its chemical inertness, biocompatibility, and ease of functionalization [15]. Its micron-sized particles can form hierarchical porous structures, while nanoscale particles provide a higher specific surface area [16].

3-Aminopropyltriethoxysilane (APTES) is an important silane coupling agent with two active functional groups in its molecular structure: amino (-NH_2_) and ethoxy (-OCH_2_CH_3_) [17]. This unique bifunctional characteristic endows it with extensive application value in the field of materials science. Proença et al. [18] utilized APTES as a silane self-assembled monolayer to functionalize a nanoplasmonic Au: CuO thin film. The terminal -NH_2_ groups on APTES form hydrogen bonds with carbon monoxide (CO) molecules, leading to their effective capture and concentration on the sensing interface. This molecular-level process markedly improves the detector’s overall responsiveness and specificity, yielding an approximately threefold enhancement in signal output when exposed to 50 ppm CO. Divyamani et al. [19] employed APTES as a crosslinking agent to modify ZnO electrodeposited on a glassy carbon electrode. The molecular structure of APTES allows it to graft onto the ZnO surface covalently at its silane end and simultaneously immobilize the cortisol antibody through its amino terminus. This specific arrangement is instrumental in achieving precise and sensitive cortisol detection. As a result, employing APTES as a functionalizing agent markedly boosts the adsorption capacity at the interface of gas-sensitive layers and the molecules of interest.

This study employs a one-pot method to synthesize ordered mesoporous SiO_2_ nanoparticles (MSNs) with uniform size and distinct structure. Under weak alkaline conditions, self-polymerization facilitates the modification of APTES onto the surface of MSNs. Sensors constructed based on the synthesized materials demonstrate that APTES-modified MSNs exhibit high sensitivity, short response/recovery time, excellent selectivity, and strong stability, with NH_2_-MSNs-2 showing the optimal gas-sensitive performance. Through systematic analysis of various characterization data, combined with experimental validation and theoretical calculations, the sensing mechanism of the materials toward 3H2B is elucidated, providing scientific explanations for the differences in gas-sensitive properties among the materials.

## 2. Materials and Methods

### 2.1. Reagents and Instruments

All chemical reagents were used as received without further purification. Tetraethyl orthosilicate (TEOS ≥ 98%) and 3-aminopropyltriethoxysilane (APTES, ≥98%) were purchased from Sigma-Aldrich Co., Shanghai, China, and Aladdin Co, Shanghai, China, respectively. Cetyltrimethylammonium bromide (CTAB ≥ 99%) was obtained from Sinopharm Chemical Reagent Co., Ltd., Shanghai, China Ammonia solution (97.0%), hydrochloric acid (37 wt%), ethyl ether (≥99.7%), and anhydrous ethanol (≥99.7%) were acquired from. The *Listeria monocytogenes* strain CMCC54002, purchased from Shanghai Beikangnuo Biotechnology Co., Ltd., Shanghai, China, belongs to serotype 1/2a. This serotype is one of the most globally prevalent serotypes associated with foodborne illnesses (listeriosis), which are caused by this pathogen.

### 2.2. Synthesis of NH_2_-MSNs via a Sol–Gel One-Pot Method

NH_2_-MSNs were synthesized via a sol–gel one-pot method using water, ethanol, and ether as cosolvents, CTAB as the surfactant, ammonia water as the catalyst, and TEOS and APTES as co-condensing silanes. In a typical procedure, 0.5 g of CTAB was dissolved in an emulsion system composed of 70 mL of H_2_O, 0.8 mL of ammonia water, 15 mL of ether, and 5 mL of ethanol. The mixture was stirred at 1000 rpm for 30 min at 15 °C, after which a mixture of 2.5 mL TEOS and 0.1 mL APTES was rapidly added dropwise to the above mixture. The resulting mixture was vigorously stirred at 1000 rpm for 4 h at 15 °C. Subsequently, 1 mL of HCl (37%) was added to terminate the base-catalyzed reaction. A white precipitate was obtained by centrifugation at 4200 rpm for 12 min, washed with distilled water, and dispersed in ethanol.

The as-prepared product was purified by gradient centrifugation. First, the product (undried) was dispersed in ethanol (100 mL) and subjected to ultrasonic dispersion for 30 min at an ultrasonic power of 330 W. Then, different centrifugation speeds (1500, 2000, 2500, 3000, 3200, and 3500 rpm) were investigated. When the particle suspension was treated by first centrifuging at 3000 rpm for 3 min and then at 3200 rpm for 3 min, large particles and aggregates could be effectively removed. The resulting nanoparticle-containing suspension was centrifuged at 4200 rpm for 12 min to obtain purified nanoparticles. The purified nanoparticles were stored in ethanol or dried in air at 60 °C for analysis.

The CTAB template within the nanoparticles was removed via an extraction method using 15 mL of HCl (37%) in 120 mL of ethanol at 70 °C for 24 h under stirring. The final nanoparticles were obtained by centrifugation at 4200 rpm for 12 min and washed three times with water. The purified and extracted nanoparticles (NH_2_-MSNs) were dried in air at 60 °C and then sealed for analysis and characterization.

When the volume ratios (r) of ethanol/ether were 0.5 (10 mL/20 mL), 1 (20 mL/20 mL), and 1.5 (30 mL/20 mL), the resulting silica samples were designated as NH_2_-MSNs-1, NH_2_-MSNs-2, and NH_2_-MSNs-3, respectively.

### 2.3. Characterization Methods and Instruments

The following characterization methods were employed to analyze the synthesized materials: X-ray diffraction (XRD, Bruker D4, Bruker, Karlsruhe, Germany) was used to analyze the composition and phase structure of the materials. The analysis was conducted at room temperature using Cu-Kα (λ = 0.15418 nm) radiation within the 2θ range of 5–80°. Scanning Electron Microscopy (SEM, Zeiss Gemini SEM S00, Zeiss, Jena, Germany), transmission electron microscopy (TEM), and high-resolution transmission electron microscopy (HRTEM, TECNAI G2 F20, FEI, Hillsboro, OR, USA) were used for electron microscopy. The textural properties of the samples were characterized using Brunauer–Emmett–Teller (BET, ASAP 2460, Micromeritics, Norcross, GA, USA) and Barre–Joyner–Halenda (BJH) analyses to determine specific surface area, pore volume, and pore size distribution. Surface chemical composition was analyzed via X-ray photoelectron spectroscopy (XPS, AXIS ULTRA DLD, Kyoto, Japan), while surface reaction mechanisms were investigated using Fourier Transform Infrared spectroscopy (FTIR, Bruker Tensor 27, Bruker, Ettlingen, Germany). Quartz Crystal Microbalance (QCM) tests were performed using a frequency counter/timer (Keysight 53230A, Keysight Technologies, Shanghai, China).

### 2.4. Procedure for QCM Sensor Fabrication and Testing

The QCM electrodes were sequentially cleaned with acetone and deionized water for 30 min each, dried with high-purity nitrogen, and then surface-modified. The fundamental frequency was determined by recording the corresponding resonant frequency. The as-prepared material was mixed with deionized water to form a 6 mg mL^−1^ suspension. A 1.5 μL aliquot of the suspension was dropped onto the silver surface of the QCM electrode to form a thin film at room temperature. Finally, a 7.04 L plastic chamber was constructed in the laboratory to store the prepared QCM sensors. The sensing experiments in this study were conducted under a relative humidity of 60 ± 1% and at a room temperature of 25 ± 1 °C throughout the entire testing process. A precisely calculated volume of the target liquid was injected into a heating plate using a microliter syringe. With the assistance of a fan, the liquid rapidly vaporized, allowing the analytical gas flow to be introduced until the sensor response stabilized. The testing process is shown in Figure S1. To further investigate the sensing mechanism of the NH_2_-MSNs-2 material, gas-sensing tests were conducted at two different temperatures, and the corresponding enthalpy change was derived by processing the data using the Clausius–Clapeyron equation.

### 2.5. Practical Sample Analysis of LM

The strain was preserved in a glycerol stock at −80 °C. To begin the experiment, the bacteria were activated by an overnight culture in BHI broth. A series of ten-fold dilutions of the bacterial suspension was then prepared, yielding concentrations from 10^1^ to 10^7^ CFU mL^−1^. For each dilution, 150 μL was transferred to a 40 mL headspace vial and incubated at 37 °C with shaking at 150 rpm. The growth of these cultures, initiated at different bacterial densities, was monitored by sampling every 2 h for analysis using the NH_2_-MSNs-2 sensors. Concurrently, to estimate the bacterial density, 200 μL aliquots from the same set of dilutions were added to a 96-well plate for optical density (OD_600_) measurement. For comparative purposes, *Staphylococcus aureus* and *Escherichia coli* were also cultivated and analyzed following the same protocol.

### 2.6. Statistical Analysis

All experiments were conducted with a minimum of three independent biological replicates (*n* = 3) to ensure reproducibility and statistical robustness. Quantitative data are presented as mean ± standard deviation, calculated from the triplicate measurements. Error bars in all figures represent the SD derived from these independent experiments.

## 3. Results

### 3.1. Characterizations of Materials

**Figure 1:**
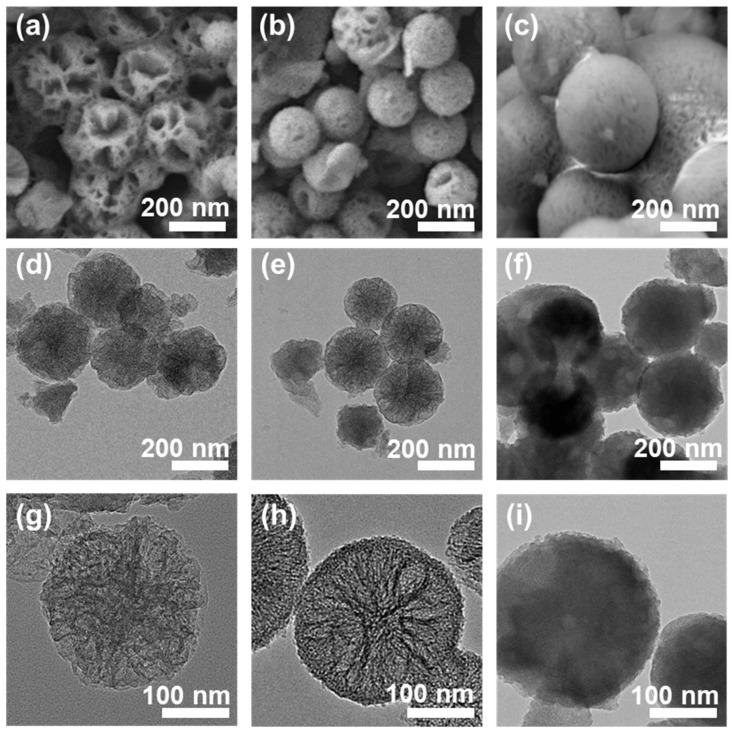
Scanning electron microscope (SEM) images (**a**–**c**) and transmission electron microscope (TEM) images (**d**–**i**) of MSNs under different ethanol/ether ratios.

Through systematic regulation of the ethanol-to-ether volume ratio, a series of mesoporous SiO_2_ nanospheres were successfully synthesized under ambient conditions. SEM characterization (Figure 1) demonstrated that the ethanol/ether ratio significantly modulates the morphological evolution of these nanospheres:

At an ethanol/ether volume ratio of 0.5, the NH_2_-MSNs-1 sample exhibited an average diameter of approximately 180 nm, featuring a nascent spherical structure with prominent porous surface characteristics (Figure 1a,d,g). When the ratio increased to 1.0, the NH_2_-MSNs-2 sample displayed an expanded diameter of ~200 nm, with enhanced mesoporous structural definition, high-density uniform distribution, relatively smooth surface, and well-ordered spherical morphology (Figure 1b,e,h). TEM analysis further corroborated the presence of large mesoporous channels, as evidenced by the distinct contrast variations between dark and light regions within the nanosphere interior (Figure 1). At an ethanol/ether ratio of 1.5, the NH_2_-MSNs-3 sample achieved a diameter of ~250 nm, accompanied by a marked improvement in surface smoothness (Figure 1c,f,i). Comprehensive analysis reveals that the ethanol/ether ratio serves as a critical regulator in the formation of MSNs: elevated ethanol concentrations induce the formation of surface branching structures while preserving mesoporous structural integrity [20]. This morphological evolution pattern elucidates the precise regulatory mechanism underlying solvent ratio-dependent self-assembly processes, providing crucial experimental insights for the rational design of mesoporous materials with tailored pore architectures and surface topographies [21].

In the magnified region of the TEM image for NH_2_-MSNs-2, spatial coexistence of white dot-like features and stripe-like features is observed within the area demarcated by red dashed lines (Figure S2). Through high-resolution imaging analysis, the white dot-like features correspond to cross-sectional views of nanoscale mesopore channels within the silica framework, while the stripe-like features reflect the directional extension of mesoporous channels [16]. The coexistence of these distinct morphological signatures further demonstrates that the mesoporous structures formed under this synthetic condition exhibit spatial isotropy in distribution, with both mesopore diameter and pore connectivity achieving a high degree of uniformity [22]. This microstructural characterization provides direct evidence for the homogeneous integration of mesopores within the silica matrix, supporting the precise control of solvent-mediated self-assembly processes in the rational design of mesoporous nanomaterials.

**Figure 2:**
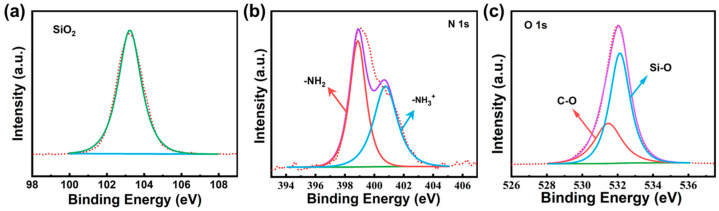
High-resolution XPS spectra of (**a**) Si 2p, (**b**) N 1s and (**c**) N 1s for NH_2_-MSNs-2.

FTIR was employed to systematically elucidate the chemical functional groups on NH_2_-MSNs (Figure S3). The broad absorption band observed in the 853–1088 cm^−1^ range is attributed to the asymmetric stretching vibration of Si-O-Si bonds, resulting from the condensation polymerization of Si-OH groups to form cyclic or linear silica network structures [23]. A characteristic Si-O bending vibration peak at 464 cm^−1^ further confirms the presence of SiO_2_ crystalline phases. The vibration peak at 2925 cm^−1^ corresponds to the alkyl chains (-CH_2_-) of (3-aminopropyl)triethoxysilane (APTES), providing direct evidence of successful silane coupling agent grafting [24]. Additionally, the 1628 cm^−1^ peak arises from the bending vibration of physically adsorbed water molecules, indicating surface hydrophilicity [25]. Complementary XPS analysis offers molecular-level validation of the functionalization mechanism. As shown in Figure 2a, the Si 2p spectrum exhibits a pronounced peak at 103.2 eV, characteristic of Si-O bonds in silica frameworks. The N 1s spectrum (Figure 2b) reveals dual components: the 401.5 eV peak corresponds to primary amine groups (-NH_2_, R-NH_2_) and the 399.4 eV peak to secondary amine groups (-NH-, R-NH-R), confirming successful amino group incorporation with chemical environment heterogeneity [23]. Deconvolution of the O 1s spectrum (Figure 2c) for NH_2_-MSNs-2 shows two resolved peaks at approximately 530.0 eV (C-O bonds from residual ethoxy groups of APTES) and 532.0 eV (Si-O bonds in the silica matrix), directly verifying the formation of organic-inorganic hybrid structures [26]. These multi-technique characterization results collectively demonstrate the controlled functionalization of APTES onto SiO_2_ surfaces, achieving a composite nanosystem that integrates ordered mesoporous architecture with chemically active amino groups [27]. The precise correlation between spectroscopic signatures and material composition provides robust experimental evidence for solvent-mediated self-assembly processes in the rational design of tailored mesoporous nanomaterials.

**Figure 3:**
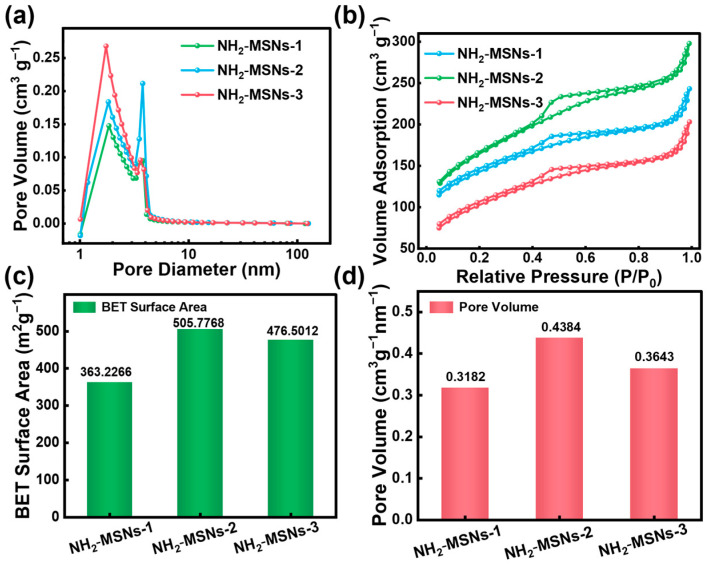
(**a**) Corresponding pore size distribution curves and (**b**) N_2_ adsorption/desorption isotherms of NH_2_-MSNs-1, NH_2_-MSNs-2, and NH_2_-MSNs-3, (**c**) BET surface area, and (**d**) total pore volume of NH_2_-MSNs-1, NH_2_-MSNs-2, and NH_2_-MSNs-3.

The preservation of highly ordered mesoporous structures after functionalization is demonstrated by Synchrotron X-ray Diffraction (SXRD) in Figure S4. The patterns for NH_2_-MSNs-1, NH_2_-MSNs-2, and NH_2_-MSNs-3 all confirm a well-defined long-range order, indicating that the functionalization process does not compromise their structural integrity. Nitrogen adsorption-desorption isotherm analysis was systematically employed to investigate the pore structural characteristics of the NH_2_-MSNs series samples. The experimental results reveal that NH_2_-MSNs-1, NH_2_-MSNs-2, and NH_2_-MSNs-3 all exhibit typical Type IV isotherm behavior accompanied by H1-type hysteresis loops (Figure 3a,b), which are characteristic of well-ordered mesoporous materials with uniform, cylindrical pores [28]. The pore size distribution curves for all samples are concentrated within the 2–5 nm range, conforming to the definition of mesoporous structures. Further analysis using the Barrett–Joyner–Halenda model allowed for the determination of average pore diameters and pore volumes. As shown in Figure 3c, BET surface area analysis demonstrates that NH_2_-MSNs-2 exhibits the most outstanding surface properties, with a specific surface area of 505.77 m^2^ g^−1^, significantly exceeding those of NH_2_-MSNs-3 (476.50 m^2^ g^−1^) and NH_2_-MSNs-1 (363.22 m^2^ g^−1^). Pore volume analysis (Figure 3d) corroborates this trend: NH_2_-MSNs-2 shows a pore volume of 0.43 cm^3^ g^−1^, outperforming NH_2_-MSNs-3 (0.36 cm^3^ g^−1^) and NH_2_-MSNs-1 (0.31 cm^3^ g^−1^). This variation in structural parameters is highly correlated with the morphological evolution of the materials—NH_2_-MSNs-2, synthesized at an ethanol/ether ratio of 1.0, forms a uniform mesoporous structure (200 nm diameter) with smooth surface characteristics, thereby providing enhanced surface area and pore volume. Comprehensive characterization data (Table S1) indicate that solvent ratio regulation not only influences nanosphere diameter and surface morphology but also directly determines the pore channel parameters of the mesoporous structures. The superior pore characteristics of NH_2_-MSNs-2 originate from its unique self-assembly process: an optimal ethanol concentration promotes the uniform condensation of silicate precursors while maintaining the connectivity of mesoporous channels, ultimately yielding an ordered mesoporous structure with both high surface area and regular pore channels [29]. This structure-property relationship provides definitive experimental evidence for optimizing the gas adsorption performance of mesoporous materials and validates the effectiveness of the solvent ratio as a critical regulatory parameter in the synthesis of mesoporous SiO_2_ nanospheres.

### 3.2. Gas Sensitivity Performance

**Figure 4:**
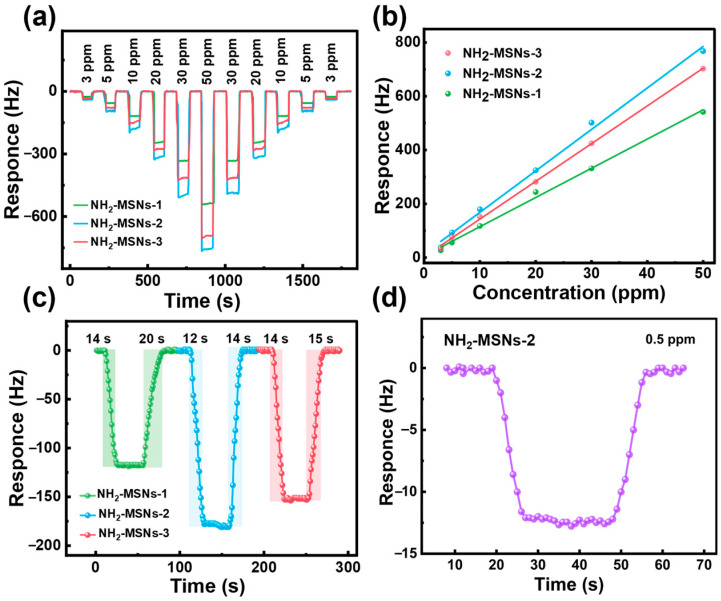
(**a**) dynamic responses to 3H2B (3–50 ppm) (**b**) relationship between responses and 3H2B concentration (3–50 ppm); (**c**) response/recovery time of the sensors to 3H2B (10 ppm)of NH_2_-MSNs-1, NH_2_-MSNs-2 and NH_2_-MSNs-3. (**d**) NH_2_-MSNs-2’s response and recovery curve for 3H2B at 500 ppb.

Dynamic response analysis via QCM (Figure 4a,b) systematically investigated the gas-sensitive properties of mesoporous SiO_2_ with distinct morphologies toward 3H2B (concentration range: 3–50 ppm). Experimental results demonstrate that all prepared QCM gas sensors exhibit instantaneous response characteristics upon exposure to the target gas and rapidly recover to the baseline state in ambient air. These superior response-recovery kinetic properties provide technical feasibility for non-destructive, rapid detection of *LM* in food safety applications. Further analysis reveals a strict positive correlation between sensor response values and 3H2B concentration, with high reversibility maintained across multiple cyclic tests, confirming excellent material repeatability and stability. Notably, the NH_2_-MSNs-2-based QCM sensor demonstrates the highest sensitivity under dynamic concentration gradients, showing significantly elevated response values compared to other samples. This performance superiority is attributed to its optimized mesoporous architecture, characterized by a uniform 200 nm diameter and a high specific surface area (505.77 m^2^ g^−1^), which synergistically enhances rapid gas molecule diffusion and efficient adsorption. Critically, within the 3–50 ppm concentration range, all three sensors exhibit exceptional linear relationships between response values and target gas concentration (Table S2), with linear correlation coefficients exceeding 0.99. This verifies the precise quantitative analysis capability of NH_2_-MSNs-based QCM sensors for 3H2B detection. The structure-property relationship highlights how the regulation of mesoporous morphology significantly impacts gas sensing performance, providing key experimental evidence for the development of highly selective and sensitive biomarker detection devices [30]. Comprehensive characterization validates that solvent ratio regulation strategies effectively optimize the performance of mesoporous SiO_2_-based gas sensors. The exceptional pore characteristics and linear response behavior of NH_2_-MSNs-2, in particular, highlight its strong potential for practical implementation in food safety monitoring systems. This work establishes a clear experimental foundation for the rational design of mesoporous nanomaterials with tailored gas-sensitive properties for real-world biosensing applications [31].

The response/recovery kinetics of gas sensors, defined as the time required to achieve 90% of the equilibrium state from the baseline, constitute a critical metric for evaluating their practical monitoring efficacy toward target analytes. For 10 ppm 3H2B detection, the NH_2_-MSNs-1, NH_2_-MSNs-2, and NH_2_-MSNs-3 based QCM sensors exhibit response/recovery times of 14/20 s, 12/14 s, and 14/15 s, respectively (Figure 4c). Among these, NH_2_-MSNs-2 demonstrates the most favorable kinetic performance, attributed to two synergistic factors: first, its superior specific surface area (505.77 m^2^ g^−1^) and optimized pore size distribution (2–5 nm) facilitate efficient gas molecule diffusion and adsorption/desorption dynamics; second, the weak chemical interaction between APTES-derived -NH- groups and the C=O moiety of 3H2B enhances selective adsorption while maintaining rapid recovery kinetics [32]. Further validation via Figure 4d reveals that the NH_2_-MSNs-2 sensor achieves a remarkable detection limit of 13 Hz at 0.5 ppm 3H2B, underscoring its exceptional sensitivity in low-concentration regimes. This performance superiority stems from the precise structural tuning achieved through solvent ratio optimization—the 1.0 ethanol/ether ratio fosters a balanced mesoporous architecture that maximizes active site accessibility while preserving structural integrity. The quantitative correlation between response magnitude and analyte concentration (R^2^ > 0.99) further confirms the sensor’s reliability for precise biomarker quantification. Collectively, these results establish NH_2_-MSNs-2 as an optimal candidate for food safety monitoring systems, particularly for the non-invasive detection of *LM* biomarkers. The demonstrated structure-property relationship provides a rational design paradigm for next-generation mesoporous nanomaterials with tailored gas-sensitive properties, highlighting the critical role of solvent-mediated self-assembly in achieving high-performance biosensing platforms.

**Figure 5:**
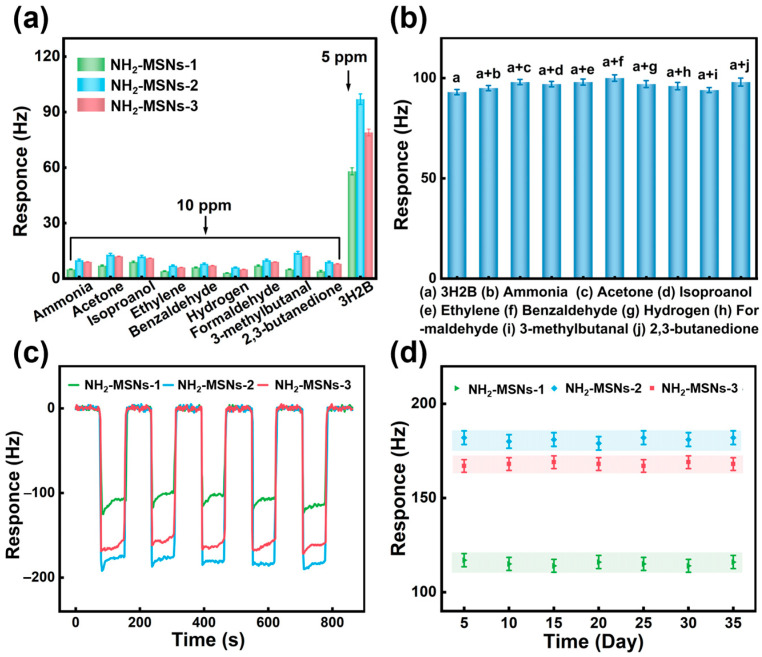
(**a**) Selectivity of NH_2_-MSNs-1, NH_2_-MSNs-2, and NH_2_-MSNs-3-based sensors (5 ppm 3H2B and 10 ppm other interfering gases); (**b**) identification tests of NH_2_-MSNs-2 sensors for gas mixtures containing 5 ppm 3H2B and 10 ppm other interfering gases. Repeatability of (**c**) 3H2B (10 ppm) and (**d**) long-term stability of NH_2_-MSNs-1, NH_2_-MSNs-2, and NH_2_-MSNs-3 based sensors and different concentrations of polydopamine.

The selective discrimination of target MVOCs from environmental interferents is paramount for accurate analysis. To evaluate the selectivity of NH_2_-MSNs-1, NH_2_-MSNs-2, and NH_2_-MSNs-3-based sensors, systematic tests were conducted at room temperature using 5 ppm target gas (3H2B) and 10 ppm interfering gases. The interfering gases included common atmospheric components (formaldehyde, ethanol, acetone, H_2_S) and *LM*-characteristic MVOCs (2,3-butanedione, 3-methylbutanal, benzaldehyde, 2,5-dimethylpyrazine). Even at the low concentration of 5 ppm, the NH_2_-MSNs-2 sensor demonstrated a significantly higher response to 3H2B, with response intensity at least threefold greater than that of all tested interferents (Figure 5a). To further assess anti-interference capability, the sensor was exposed to 10 ppm of each interfering gas. As shown in Figure 5b, the response fluctuations remained within ±15%, conclusively verifying the outstanding selectivity of the NH_2_-MSNs-2-based sensor. Collectively, these results confirm that the NH_2_-MSNs-2 sensor combines high sensitivity to 3H2B with robust resistance to common interferents, making it highly promising for complex environmental gas detection applications. Reproducibility, a critical metric for sensor reliability in real-world samples, was evaluated through five consecutive cycles of exposure to 10 ppm 3H2B. The consistent response-recovery profiles (Figure 5c) with no significant degradation confirm excellent reproducibility across all three sensor variants. Long-term stability tests (Figure 5d) revealed negligible response variation (<5%) for the NH_2_-MSNs-2 sensor over 30 days when exposed to 10 ppm 3H2B, further validating its superior durability. Humidity tests (Figure S5) demonstrated minimal frequency shift (<10%) under varying relative humidity conditions, indicating that water vapor interference is substantially weaker than the response to 3H2B. This differential sensitivity arises from the higher molecular weight and stronger positive inductive effect of 3H2B compared to water molecules, which enhances its selective adsorption on the APTES-functionalized mesoporous surface. These comprehensive characterizations establish NH_2_-MSNs-2 as an optimal sensing platform that integrates high sensitivity, selectivity, reproducibility, and long-term stability. The demonstrated structure-property relationships provide a rational design blueprint for next-generation MVOC sensors.

### 3.3. Gas-Sensing Mechanism

Thermodynamic investigation of adsorption processes in mass-sensitive gas sensors is rigorously conducted within the framework of classical physicochemical adsorption theory, utilizing the Clausius–Clapeyron equation in synergy with temperature-dependent adsorption experiments to quantitatively characterize the enthalpy change (ΔH) during target gas molecule-sensing material interactions [33]. This approach facilitates a fundamental mechanistic understanding by elucidating the thermodynamic nature of adsorption behavior, where the magnitude of ΔH serves as a critical parameter for distinguishing physisorption, chemisorption, and optimal weak chemisorption regimes that balance selectivity with reversible adsorption-desorption kinetics [34].

**Figure 6:**
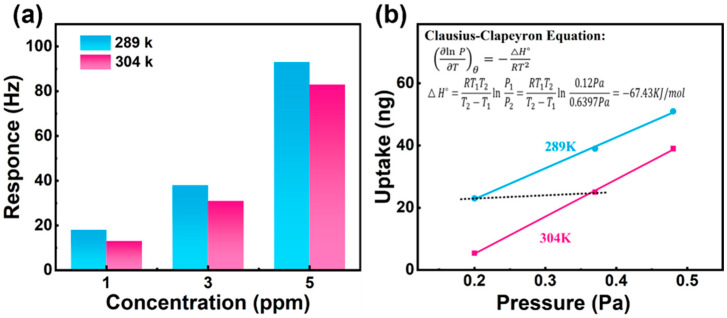
(**a**) Sensing value of 3H2B vapor by NH_2_-MSNs-2 at different concentrations at 289 k and 304 k; (**b**) two isotherms of enthalpy change (−ΔH) based on experimental data in (**a**).

The magnitude of ΔH categorizes adsorption regimes: when ΔH > −40 kJ·mol^−1^, adsorption is dominated by reversible physisorption, ensuring rapid response-recovery kinetics; for ΔH < −80 kJ·mol^−1^, irreversible chemisorption occurs (involving chemical bond formation), which enhances selectivity but compromises reversibility; the optimal regime (−80 kJ·mol^−1^ ≤ ΔH ≤ −40 kJ·mol^−1^) achieves a balance between selective adsorption and reversibility through weak chemisorption—moderate bonding strength enhances target molecule recognition while maintaining reversible adsorption-desorption cycles [35]. As demonstrated in Figure 6a,b, temperature-dependent experiments combined with the Clausius–Clapeyron equation yield a ΔH value of −67.43 kJ·mol^−1^ for the interaction between 3H2B and NH_2_-MSNs-2, placing it within the weak chemisorption window. This value validates the material design rationale: weak hydrogen bonding between APTES-functionalized -NH groups and the C=O moiety of 3H2B enhances selectivity, while moderate adsorption strength preserves reversible desorption capability [36]. Consequently, the sensor achieves high sensitivity and maintains stability across multiple cycles. This thermodynamic characterization provides theoretical justification for the reliable application of sensing materials in complex gas environments.

### 3.4. Practical Application

**Figure 7:**
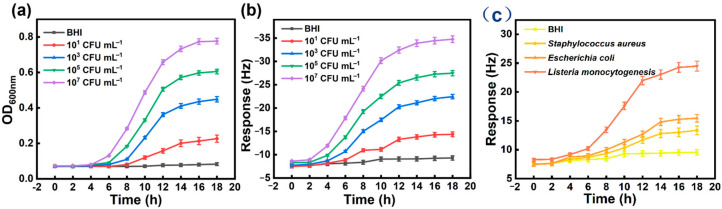
(**a**) Schematic diagram of the practical sample testing. (**b**) NH_2_-MSNs-2 sensor response and (**c**) OD values to different concentrations of *LM* (10^1^–10^7^ CFU mL^−1^).

Through systematic comparison of the performance differences between the traditional turbidity method and the QCM gas sensor functionalized with NH_2_-MSNs-2 in the detection of *LM*, this study reveals significant advantages of the latter in terms of rapidity, specificity, and biomass correlation. Based on real-time monitoring data from Figure 7a,b, the QCM sensor exhibits a typical three-stage kinetic response to the *LM* cultivation process: 0–4 h as the initial slow growth phase with a gradual increase in response signal; 4–8 h as the rapid rising phase with a significant acceleration in signal growth; and 8–12 h as the decay phase with a gradual decline in growth rate, eventually reaching a steady-state plateau after 12 h [37]. This kinetic curve shows a strong positive correlation with the *LM* growth curve measured by a microplate reader (characterized by OD_600_ absorbance values), confirming the intrinsic synchronization between the sensor response and bacterial biomass growth at the mechanistic level.

Compared to the limitations of the traditional turbidity method, which relies on OD_600_ values to indirectly reflect bacterial concentration, this sensor achieves rapid early response capability by targeted detection of 3H2B, an MVOC produced by *LM* metabolism. Experimental data show that the sensor can generate distinguishable signals when the bacterial density is below the detection threshold of the turbidity method (which typically requires 12–18 h of cultivation to observe significant changes), enabling early detection of *LM* 6–8 h earlier than traditional methods. More importantly, the traditional turbidity method suffers from the inability to distinguish between live and dead bacteria, as its OD value changes may include non-specific interference from dead bacterial debris or turbidity of the culture medium, leading to false-positive results [38,39]. In contrast, this sensor, based on the specific recognition mechanism of metabolites from live bacteria, responds only to 3H2B produced by active bacteria, ensuring that the detection results strictly correspond to the population of pathogenic live bacteria. In the blank control group (culture medium without *LM* inoculation), both the sensor response values and the microplate reader OD_600_ values show no significant changes over the same cultivation period, excluding non-specific interference from the culture medium matrix to the sensor [40].

Cross-validation experiments with *Staphylococcus aureus* and *Escherichia coli* (Figure 7c) further confirm the pathogen specificity of the sensor. Over an 18 h cultivation period, the sensor response intensity to *LM* continues to rise and stabilizes after 14 h, while the response values to the two non-target bacteria remain at baseline levels and show no significant fluctuations over time [41]. Through the functional design of the NH_2_-MSNs-2 receptor material, the sensor achieves high selective recognition of 3H2B, with a cross-response sensitivity to other non-specific MVOCs, ensuring anti-interference capability in complex food matrices [42]. The QCM gas sensor developed in this study, through the synergistic design of functionalized receptor materials and specific metabolic marker detection, constructs a novel detection platform that combines rapidity, specificity, and biomass correlation. Its excellent performance in *LM* detection provides a revolutionary, rapid detection technology solution for food microbiological safety detection, with significant clinical application value and industrialization prospects.

## 4. Discussion

This study successfully prepared SiO_2_ materials with an average diameter of 200 nm, a uniform mesoporous structure, and excellent dispersibility using a one-pot method by regulating the feed ratio. Using 3H2B as a model gas molecule, the influence mechanism of surface modification on the gas sensing performance of mesoporous SiO_2_-based QCM gas sensors was systematically investigated. The results show that the NH_2_-MSNs-2-based QCM sensor functionalized with amino groups exhibits excellent gas sensing characteristics: at a target gas concentration of 50 ppm, the response value reaches as high as 768 Hz, coupled with a rapid response/recovery time of 12/14 s, enabling precise identification and efficient capture of target molecules in complex gas environments. Further thermodynamic analysis and Clausius–Clapeyron equation fitting quantitatively calculated the adsorption enthalpy change (ΔH) of NH_2_-MSNs-2 for 3H2B to be −67.43 kJ·mol^−1^, revealing the molecular mechanism of selective recognition through weak chemical adsorption. Actual sample tests demonstrated that, compared to traditional detection methods, this sensor significantly improves the response speed to target gas signal changes and exhibits excellent specific recognition capability for *LM*. This study not only deepens the scientific understanding of the role of QCM sensors in the real-time non-invasive detection of *LM*, laying a theoretical foundation for the development of highly selective portable sensing devices, but also shows significant potential for application transformation in the field of rapid food safety detection.

## Data Availability

The original contributions presented in the study are included in the article/Supplementary Materials; further inquiries can be directed to the corresponding authors.

## Supplementary Materials

The following supporting information can be downloaded at https://www.mdpi.com/article/10.3390/foods14234151/s1. Figure S1. Experimental setup of the QCM system for gas or vapor sensing; Figure S2. Transmission electron microscope (TEM) image (a) of NH_2_-MSNs-2 and its corresponding enlarged view (b); Figure S3. FT-IR spectra of NH_2_-MSNs-1, NH_2_-MSNs-2, and NH_2_-MSNs-3; Figure S4. SXRD patterns of NH_2_-MSNs-1, NH_2_-MSNs-2, and NH_2_-MSNs-3; Figure S5. Responses of the NH_2_-MSNs-2-based sensor to 5 ppm 3-hydroxy-2-butanone at different relative humidity; Table S1: BET surface area, total pore volume, and average pore size of NH_2_-MSNs-1, NH_2_-MSNs-2, and NH_2_-MSNs-3; Table S2. The linear fit curve of NH_2_-MSNs-1, NH_2_-MSNs-2, and NH_2_-MSNs-3.

## Abbreviations

The following abbreviations are used in this manuscript:

APTES	3-Aminopropyltriethoxysilane
CO	Carbon monoxide
CTAB	Cetyltrimethylammonium bromide
ELISA	Enzyme-linked immunosorbent assay
FTIR	Fourier transform infrared spectroscopy
*LM*	*Listeria monocytogenes*
MVOC	Microbial volatile organic compound
MSNs	Mesoporous silica nanoparticles
OD600	Optical density
PCR	Polymerase chain reaction
QCM	Quartz crystal microbalance
SiO_2_	Silicon dioxide
SEM	Scanning electron microscopy
TEM	Transmission electron microscopy
TEOS	Tetraethyl orthosilicate
XPS	X-ray photoelectron spectroscopy
3H2B	3-hydroxy-2-butanone
-CH_2-_	Alkyl chains
-NH_2_	Amino
-OCH_2_CH_3_	Ethoxy
ΔH	Adsorption enthalpy change
