# Supplementary material for: Specific Identification of Listeria monocytogenes in Food Using a QCM Sensor Based on Amino-Modified Mesoporous SiO2 with Enhanced Surface-Active Capabilities
Source: Foods. 2025 Dec 3;14(23):4151. doi: 10.3390/foods14234151 (PMC12692001; doi:10.3390/foods14234151)
Supplement: Supplementary file 1 [file foods-14-04151-s001.zip › foods-3977510-supplementary.pdf]

foods

MDPI

Supplementary Material

Specific Identification of *Listeria monocytogenes* in Food Using a QCM Sensor Based on Amino-Modified Mesoporous SiO_2 with Enhanced Surface-Active Capabilities

Ziliang Fan †, Miaomiao Li †, Xingyu Wang, Haixia Zhou, Faraz Ahmed * and Yongheng Zhu *

College of Food Science and Technology, Shanghai Ocean University, Shanghai 201306, China; 18888820272@163.com (Z.F.); 17836914529@163.com (M.L.); 18616302670@163.com (X.W.); d250400135@st.shou.edu.cn (H.Z.)

* Correspondence: faraz@shou.edu.cn (F.A.); yh-zhu@shou.edu.cn (Y.Z.)

† These authors contributed equally to this work.

Figure and Table caption:

Figure S1. Experimental setup of the QCM system for gas or vapor sensing.

Figure S2. Transmission electron microscope (TEM) image (a) of NH_2-MSNs-2 and its corresponding enlarged view (b).

Figure S3. FT-IR spectra of NH_2-MSNs-1, NH_2-MSNs-2, and NH_2-MSNs-3.

Figure S4. SXRD patterns of NH_2-MSNs-1, NH_2-MSNs-2, and NH_2-MSNs-3.

Figure S5. Responses of NH_2-MSNs-2-based sensor to 5 ppm 3-hydroxy-2-butanone at different relative humidity.

Table S1. BET surface area, total pore volume, and average pore size of NH_2-MSNs-1, NH_2-MSNs-2, and NH_2-MSNs-3.

Table S2. The linear fit curve of NH_2-MSNs-1, NH_2-MSNs-2, and NH_2-MSNs-3.

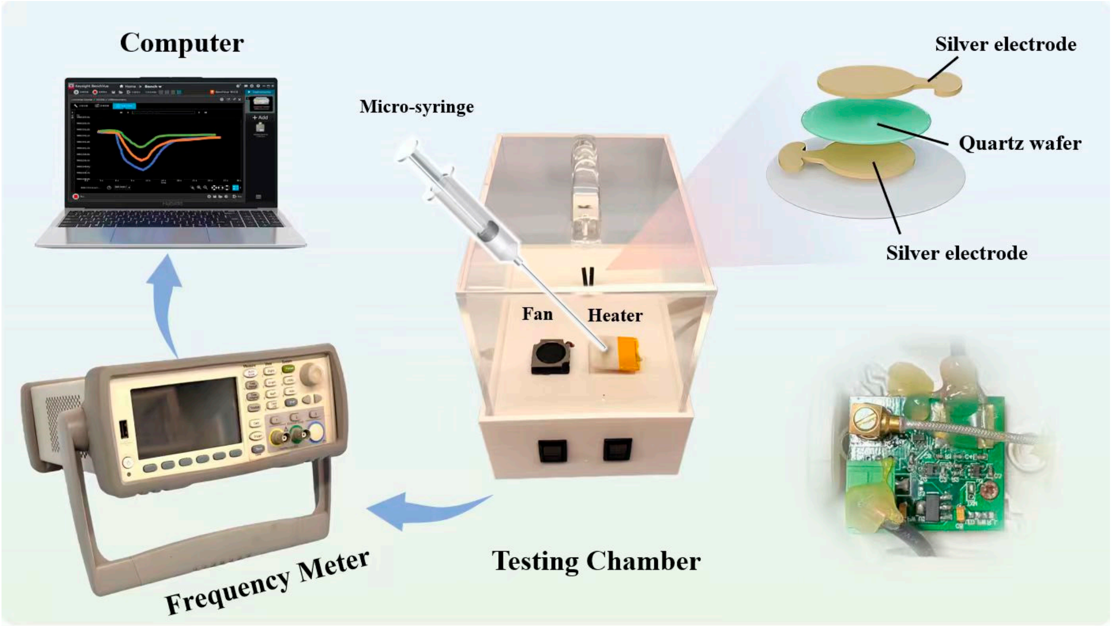

Figure S1. Experimental setup of the QCM system for gas or vapor sensing.

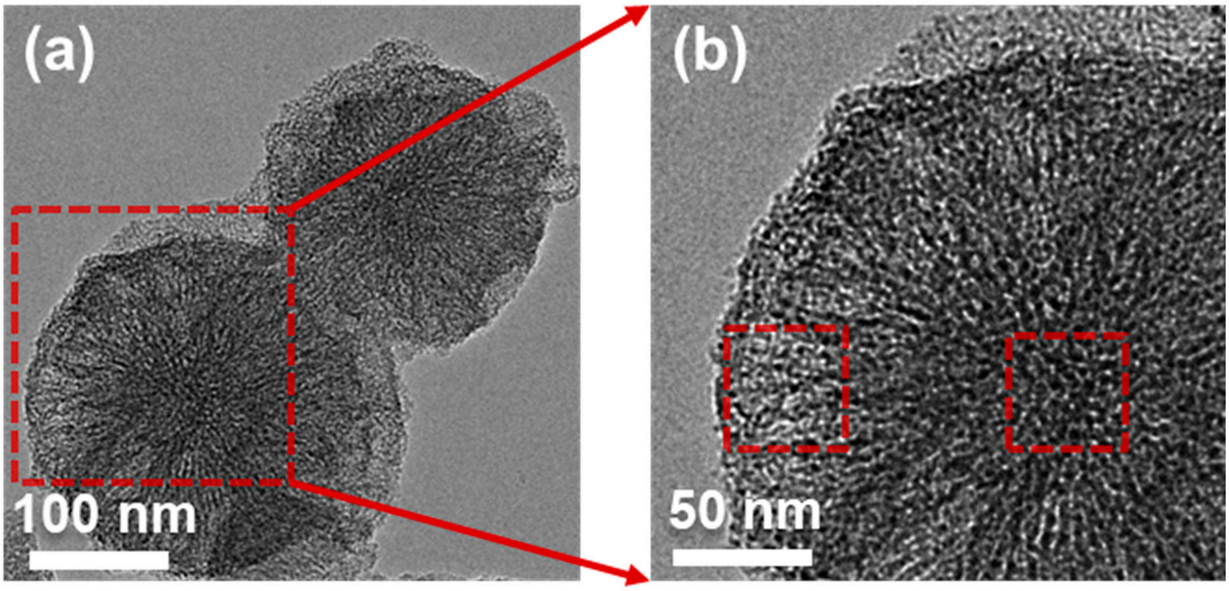

Figure S2. Transmission electron microscope (TEM) image (a) of NH_2-MSNs-2 and its corresponding enlarged view (b).

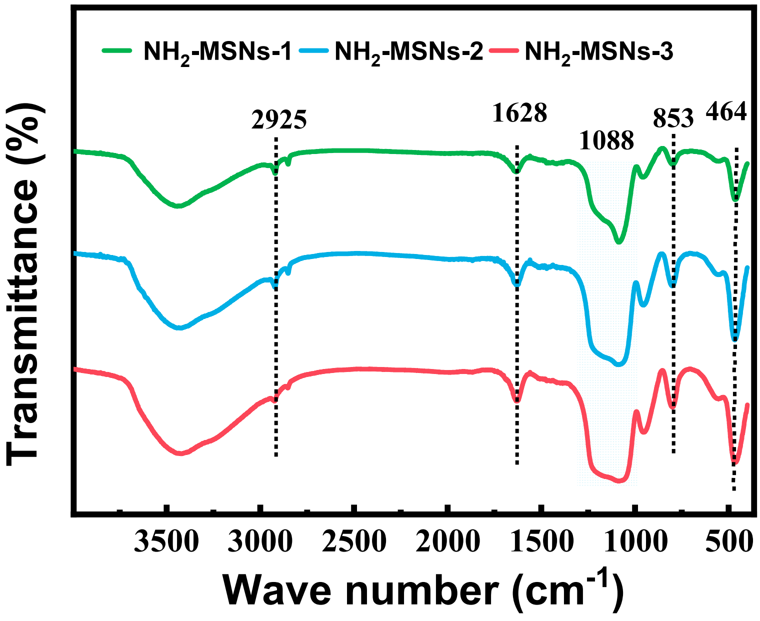

Figure S3. FT-IR spectra of NH_2-MSNs-1, NH_2-MSNs-2, and NH_2-MSNs-3.

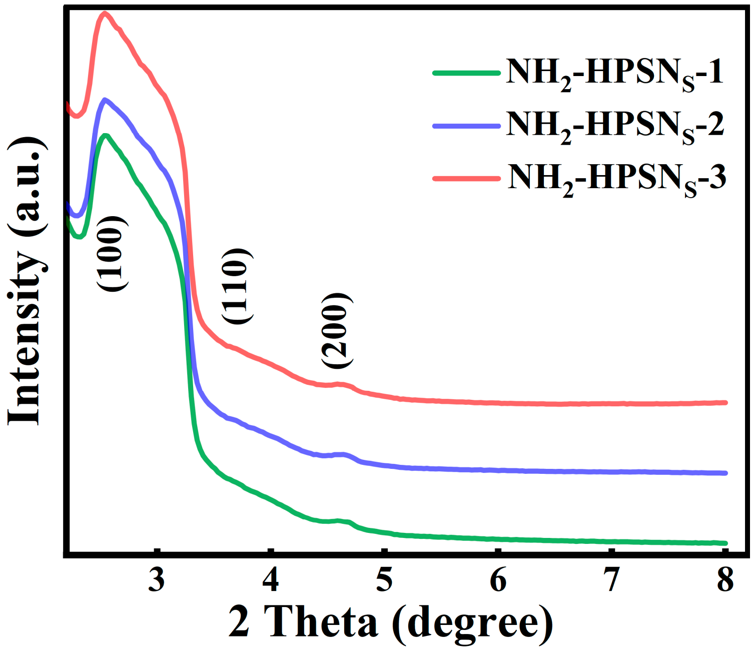

Figure S4. SXRD patterns of NH_2-MSNs-1, NH_2-MSNs-2, and NH_2-MSNs-3.

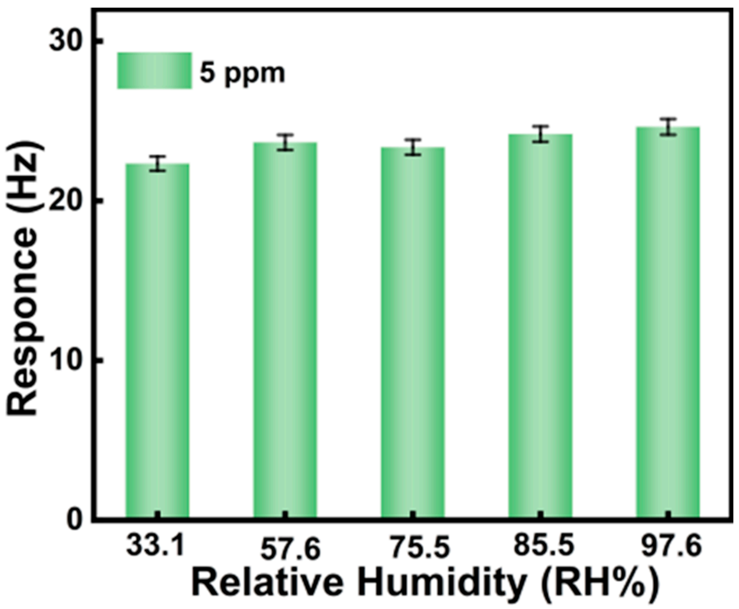

Figure S4. Responses of NH_2-MSNs-2-based sensor to 5 ppm 3-hydroxy-2-butanone at different relative humidity.

Table S1. BET surface area, total pore volume, and average pore size of NH_2-MSNs-1, NH_2-MSNs-2, and NH_2-MSNs-3.

Materials	BET surface area (m^2 g^{-1})	Pore volume (cm^3 g^{-1})	Pore size (nm)
NH_2-MSNs-1	363.23	0.32	3.58
NH_2-MSNs-2	505.78	0.44	3.01
NH_2-MSNs-3	476.50	0.37	3.36

Table S2. The linear fit curve of NH_2-MSNs-1, NH_2-MSNs-2, and NH_2-MSNs-3.

Sensing Materials	Fitting equation	R^2
NH_2-MSNs-1	y = 10.87x+6.01	0.995
NH_2-MSNs-2	y = 15.43x+13.95	0.994
NH_2-MSNs-3	y = 14.04x+2.79	0.994
